# Event and Comment

**Published:** 1908-05-15

**Authors:** 


### DENTAL REGISTER.

Vol. LXII. May 15, 1908. No. 5.

### EVENT AND COMMENT.

### Adenoids and the Dental Arch.

We reprint in this issue an article from the *Saturday Evening Post*, on the effect of post-nasal adenoid growths on the symmetrical development of the maxilla, written by the eminent specialist, Dr. Woods Hutchinson of New York. We have seldom read an article of such interest and real value to our profession, from a medical writer, and we most cordially commend its careful perusal by our readers.

We are becoming so accustomed to find malocclusion of the teeth in our patients that we have almost come to conclude that this is the normal type. An old practitioner of dentistry told us the other day of a case that had recently come under his notice of a perfect set of teeth and typically normal occlusion. He said it was absolutely the only perfect denture he had ever seen in his practice of more than thirty years. It was a real inspiration to him. It may he possible that Dr. Hutchinson takes a rather optimistic view of the matter when he says that this influence can be wholly eradicated in a generation or two by proper care of our children's noses and throats. The dental profession would be greatly pleased and encouraged if it could be assured that by any care, a reasonable percentage of this trouble properly and timely treated would result in a more normal development of the maxillary arch, and less malocclusion of the teeth. It sometimes seems a hopeless undertaking to attempt to correct all the malocclusions that come under one's observation, and in fact most practitioners avoid calling attention to these cases, for fear parents of the children may wish treatment, which the average practitioner cannot give.

We are rapidly developing the principles of scientifically correcting these defects, and a considerable number of dental practitioners have become so impressed with the need for this particular line of treatment, that they have confined their work entirely to modern orthodontia, with most gratifying results. There are, however, too few specialists to make much of an impression on the great need. We shall in the future need more rhinologists and orthodontists, unless Dr. Hutchinson's ideas are made feasible. It would also seem that a campaign of education must be inaugurated, and not only the teachers but the parents should be enlisted to insure proper remedial interference at the proper age to secure the best results. This will be another important factor in the work now being done to secure better oral hygiene for the children in our public schools and institutions. It would seem that our dentists and rhinologists are fast becoming the first guardians of the public health, and that our future duties shall be vitally concerned with the prevention rather than the reconstruction health functions. Let us read and ponder this article carefully, and determine what our future course of action in the education of our patients, at least, ought to be.

### New Ohio Examining Board and Law.

A new Board of Examiners for Ohio has just assumed office, constituted of Drs. F. H. Lyder, Pres., of Akron; F. R. Chapman, Sec., Columbus; L. L. Yonker, Bowling Green; H. C. Brown, Columbus, and W. D. Tremper, Portsmouth. The former Board made a good record, and much ought to be expected from the new one, as the men are all able, and known for their professional ideals. The new Board will have a new law to construe and enforce, which we have not had time to examine carefully or compare with the old one. The law stipulates that an examination for registration is necessary for any one who wishes to register for legal practice, but he must be a graduate of a dental college that the Board decides is reputable. It seems to us that the law should specify in general term what should constitute a "reputable dental college." There is also an opportunity for the establishment of reciprocal exchange of registration with other states having similar registration requirements on the basis of five years of ethical practice and certificate of character from the other recognized states. There is a clause which makes it possible for the Board to revoke a license for gross immoral character or behavior, certainly a desirable function, but one of questionable legality. A new clause permits dentists of other states to give clinics or demonstrations before conventions or in dental colleges—a good idea.

